# Supplementary material for: Geometric morphometric analysis of intratrackway variability: a case study on theropod and ornithopod dinosaur trackways from Münchehagen (Lower Cretaceous, Germany)
Source: PeerJ. 2016 Jun 8;4:e2059. doi: 10.7717/peerj.2059 (PMC4906676; doi:10.7717/peerj.2059)
Supplement: Figures S1 and S2 [file peerj-04-2059-s001.pdf]

Supplemental Figures S1 and S2

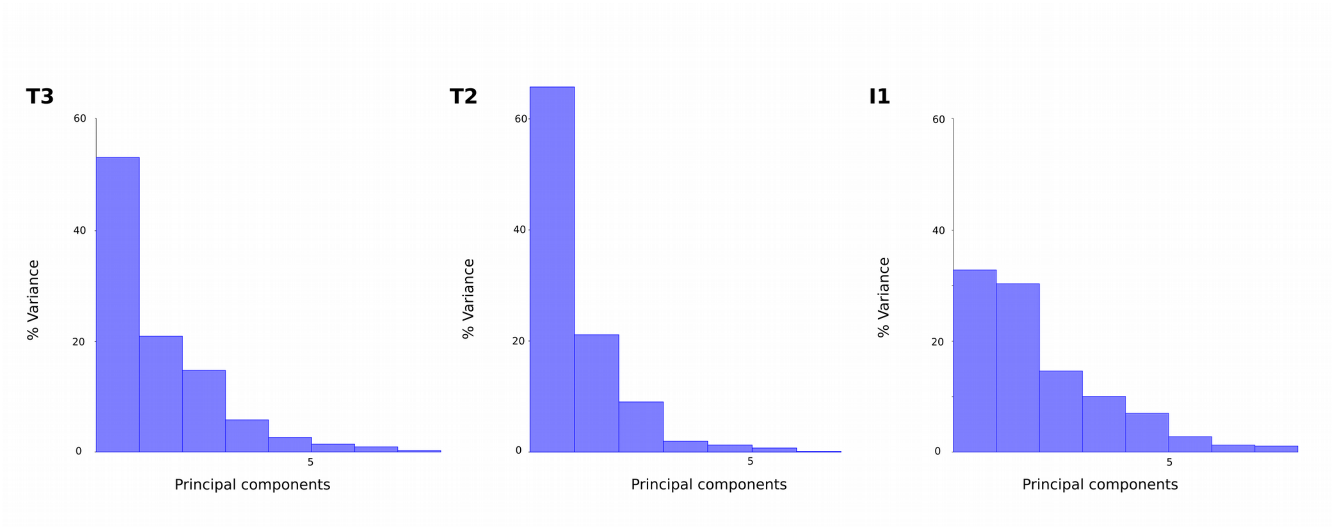

Figure S1: Loadings of the principal components of the T3, T2 and I1 trackways, based on objectively placed landmarks using the steepest slope approach. For the two theropod (T3 and T2) trackways, much of the variation is explained by the first principal component, while in the I1 trackway loadings are more equally distributed among the principal components. A similar pattern was derived from landmarks placed on interpretative outlines (compare with Fig. 9.)

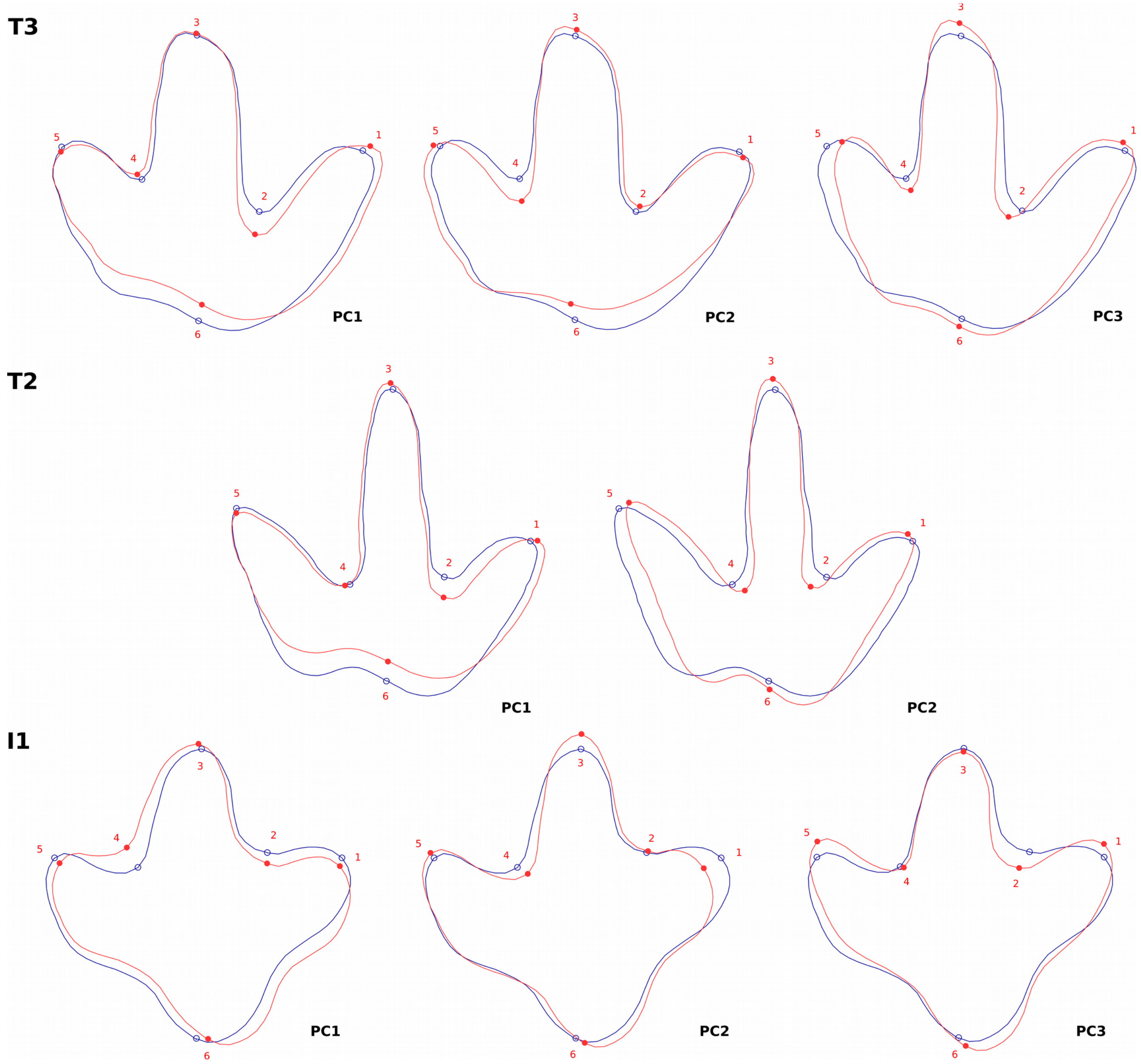

Figure S2: Shape changes described by principal components based on objectively placed landmarks using the steepest slope approach. The shape changes (red outlines, solid dots) are shown relative to the mean shapes (blue outlines, hollow dots) using warped outline drawings. Shape changes described by the first principal components of the T3 and T2 trackways are nearly identical to those derived from principal component analysis of landmarks placed on interpretative outlines; only for the I1 trackway both approaches give different results (compare with Fig. 10).
